# Association Between Vaccination Coverage Disparity and the Dynamics of the COVID-19 Delta and Omicron Waves in the US

**DOI:** 10.3389/fmed.2022.898101

**Published:** 2022-06-14

**Authors:** Diego F. Cuadros, Claudia M. Moreno, Godfrey Musuka, F. DeWolfe Miller, Phillip Coule, Neil J. MacKinnon

**Affiliations:** ^1^Digital Epidemiology Laboratory, Digital Futures, University of Cincinnati, Cincinnati, OH, United States; ^2^Department of Physiology and Biophysics, University of Washington School of Medicine, Seattle, WA, United States; ^3^ICAP at Columbia University, Harare, Zimbabwe; ^4^Department of Tropical Medicine and Medical Microbiology and Pharmacology, University of Hawaii, Honolulu, HI, United States; ^5^Department of Emergency Medicine, Medical College of Georgia, Augusta University, Augusta, GA, United States; ^6^Department of Population Health Sciences, Medical College of Georgia, Augusta University, Augusta, GA, United States

**Keywords:** COVID-19, vaccination, omicron variant, healthcare disparities, geospatial mapping

## Abstract

**Objective:**

The US recently suffered the fourth and most severe wave of the COVID-19 pandemic. This wave was driven by the SARS-CoV-2 Omicron, a highly transmissible variant that infected even vaccinated people. Vaccination coverage disparities have played an important role in shaping the epidemic dynamics. Analyzing the epidemiological impact of this uneven vaccination coverage is essential to understand local differences in the spread and outcomes of the Omicron wave. Therefore, the objective of this study was to quantify the impact of vaccination coverage disparity in the US in the dynamics of the COVID-19 pandemic during the third and fourth waves of the pandemic driven by the Delta and Omicron variants.

**Methods:**

This cross-sectional study used COVID-19 cases, deaths, and vaccination coverage from 2,417 counties. The main outcomes of the study were new COVID-19 cases (incidence rate per 100,000 people) and new COVID-19 related deaths (mortality rate per 100,000 people) at county level and the main exposure variable was COVID-19 vaccination rate at county level. Geospatial and data visualization analyses were used to estimate the association between vaccination rate and COVID-19 incidence and mortality rates for the Delta and Omicron waves.

**Results:**

During the Omicron wave, areas with high vaccination rates (>60%) experienced 1.4 (95% confidence interval [CI] 1.3–1.7) times higher COVID-19 incidence rate compared to areas with low vaccination rates (<40%). However, mortality rate was 1.6 (95% CI 1.5–1.7) higher in these low-vaccinated areas compared to areas with vaccination rates higher than 60%. As a result, areas with low vaccination rate had a 2.2 (95% CI 2.1–2.2) times higher case-fatality ratio. Geospatial clustering analysis showed a more defined spatial structure during the Delta wave with clusters with low vaccination rates and high incidence and mortality located in southern states.

**Conclusions:**

Despite the emergence of new virus variants with differential transmission potential, the protective effect of vaccines keeps generating marked differences in the distribution of critical health outcomes, with low vaccinated areas having the largest COVID-19 related mortality during the Delta and Omicron waves in the US. Vulnerable communities residing in low vaccinated areas, which are mostly rural, are suffering the highest burden of the COVID-19 pandemic during the vaccination era.

## Introduction

After almost 2 years into the COVID-19 pandemic, the B.1.1.529, Omicron SARS-CoV-2 variant was identified in South Africa on November 24, 2021, and 2 days after it was declared as a Variant of Concern (VOC) by the World Health Organization (1). Despite several efforts to contain the spread of this variant, Omicron was quickly reported in several European countries and in the US, becoming the dominant global variant driving the latest wave of the pandemic (2). The Omicron variant harbors 37 mutations in the spike protein that mediates the entry of the virus to the host cell (3). These mutations have conferred Omicron the unique ability to evade antibody neutralization while exhibiting unprecedented transmissibility (4). This genetic evolution allowed Omicron to spread around the world faster than any previous variant, outcompeting other circulating SARS-CoV-2. Omicron was first reported in the US on December 1, 2021, in California (1), and it became the variant responsible for the fourth and most severe wave in the country. New daily confirmed cases per million people under the Omicron wave tripled the highest number ever recorded during the pandemic, with 2,410 cases per million reported on January 15, 2022, compared to 756 reported on January 11, 2021 (5).

Each of the four pandemic waves has shown a characteristic epidemiological landscape determined by external forces. The first wave was mainly shaped by enforcing strict health policy measures, including lockdowns and mask mandates. In contrast, the third and fourth waves have been highly influenced by the severity and transmissibility of the emergent Delta and Omicron variants. Moreover, one of the most significant factors was implementing the vaccination campaign that started in early 2021 (6). However, the potential of massive vaccination to tackle the pandemic has been hampered by substantial heterogeneity in vaccination coverage and uptake. Some areas of the US have achieved full vaccination (people who have received two doses of the mRNA Pfizer-BioNTech or Moderna vaccines, or a single dose of the Janssen/Johnson & Johnson vaccine) in more than 80% of their population whereas other areas have less than 40% (7, 8). Vaccination coverage influences the spatial dispersion of the epidemic. In a recent publication, we showed that during the Delta wave, areas with low vaccination in the US experienced the highest rates of infection and became the pandemic's epicenter (9). We established a negative ecological association between the percentage of the vaccinated population and the number of new COVID-19 cases at the county level. Most COVID-19 cases were concentrated in the rural areas of the Western and Southern part of the country, while cases remained relatively low in the Northern region. Tracking how these external forces continue to shape the epidemic dynamics within distinct populations and communities is essential to understand the local differences in the transmission of the virus and the severity of health outcomes.

Higher transmissibility of the Delta variant compared to the previous Alpha variant along with its higher hospitalization and mortality rate shaped the dynamics of the third wave, whereas the high transmissibility of the Omicron variant, along with its ability to evade neutralizing antibodies, even those induced by vaccination, are forces that have strongly influenced the epidemic dynamic of the fourth wave. Thus, we conducted a geospatial and data visualization analysis to measure the effect of vaccination coverage disparity in the US during the fourth Omicron wave. Moreover, by comparing the epidemiological dynamics of the Delta and Omicron waves, we show how uneven vaccination coverage is influencing the local landscape of the pandemic. Refining the impact of vaccination coverage disparities under the epidemic conditions of past and present transmission waves will help to identify the most vulnerable communities with the highest health needs in the country that might suffer the highest impact during the emergence of new variants and potential upcoming waves.

## Materials and Methods

### Data Sources

We implemented a similar methodology used in a previous study aimed to assess the association of the heterogeneous distribution of vaccination coverage with the dynamics of COVID-19 during the third wave of the pandemic in the US (9). Briefly, we focused our study in the growing phase of both the Delta and Omicron waves in the US, as a result, we used data for COVID-19 cases and related mortality at the county level from July 1, 2021 to August 31, 2021, and from December 1, 2021, to January 31, 2022, obtained from Johns Hopkins University (5). Data for cumulative full vaccination rates in the total population at a county level were obtained from the CDC COVID data tracker for the contiguous US (10). We excluded the states of Colorado, Georgia, Texas, Virginia, and West Virginia due to incomplete or unreliable vaccination data. Counties were classified as rural or urban based on the 2013 National Center for Health Statistics (11, 12). As a result, data from 2,417 counties were included in the analysis. Temporal changes in COVID-19 incidence rate (i.e., new cases per 100,000 people in each time interval) and COVID-19 related mortality rate (i.e., new deaths per 100,000 people in each time interval) was estimated by generating 4-time intervals of equal length for each wave, July 1–15, July 16–31, August 1–15, and August 16–31, for the Delta wave; and December 1–15, December 16–31, January 1–15, and January 16–31, for the Omicron wave. Data were also grouped in two time periods for waves comparisons, with aggregated data from July 1 to August 31 corresponding to the Delta wave, and aggregated data from December 1 to January 31 corresponding to the Omicron wave. Cumulative vaccination rates for each time interval were estimated for the last day of each interval. Vaccination rates were aggregated in 4 groups: <40%, 40% to <50%, 50 to 50%, and >60% for visual data analysis. Institutional review board approval and informed consent were not necessary for this cross-sectional study because all data were deidentified and publicly available (Common Rule 45 CFR §46). This study follows the Strengthening the Reporting of Observational Studies in Epidemiology (STROBE) reporting guideline.

### Ecological Data Visualization Analysis

We conducted several visualization data and comparison analyses among the different periods. First, we created bivariate scatterplots illustrating associations between COVID-19 incidence and vaccination rates and COVID-19 related mortality and vaccination per county for each period in each wave. Second, we calculated the incidence and mortality rate during each wave's entire period of study and aggregated the data using the vaccination rate groups previously described. Bar charts illustrating these estimations were created, and rate ratios for incidence and mortality between high (>60%) and low (<40%) vaccination rates and 95% confidence intervals (CI) were estimated using the *rateratio.test* function in the statistical software environment R (13). Case-fatality ratios were also calculated for each vaccination group in each wave and plotted as multiple variable plots using the GraphPad software.

### Geospatial Data Analysis

The geospatial structure of the pandemic during both waves in the US was assessed using spatial bivariate and multivariate analysis using the geospatial GeoDa environment (14). First, spatial correlations between vaccination rate and incidence or mortality rate were identified using bivariate local indicators of spatial association (LISA). The bivariate LISA statistics identified significant spatial clustering based on the degree of linear association between the vaccination rate at a given location and the incidence (or mortality) rate at neighboring locations (15). Maps were generated illustrating the locations with statistically significant associations and the type of spatial association between vaccination and incidence (or mortality) rate estimations (i.e. high–high, low–low, low–high, and high-low). Second, multivariable spatial associations between all three variables, vaccination, incidence, and mortality rate, were estimated using K-means clustering analysis. K-means is a partitioning clustering method in which the data are partitioned into *k* groups (i.e., fourth groups). In this clustering method, the *n* observations are grouped into *k* clusters such that the intra-cluster similarity is maximized (or dissimilarity minimized), and the between-cluster similarity minimized (or dissimilarity maximized). A further detailed description of these geospatial methods can be found elsewhere (16, 17). Rates for each variable vaccination, incidence, and mortality in each cluster identified were reported, and maps of the results were generated using ArcGIS Pro (18).

## Results

**Figure 1:**
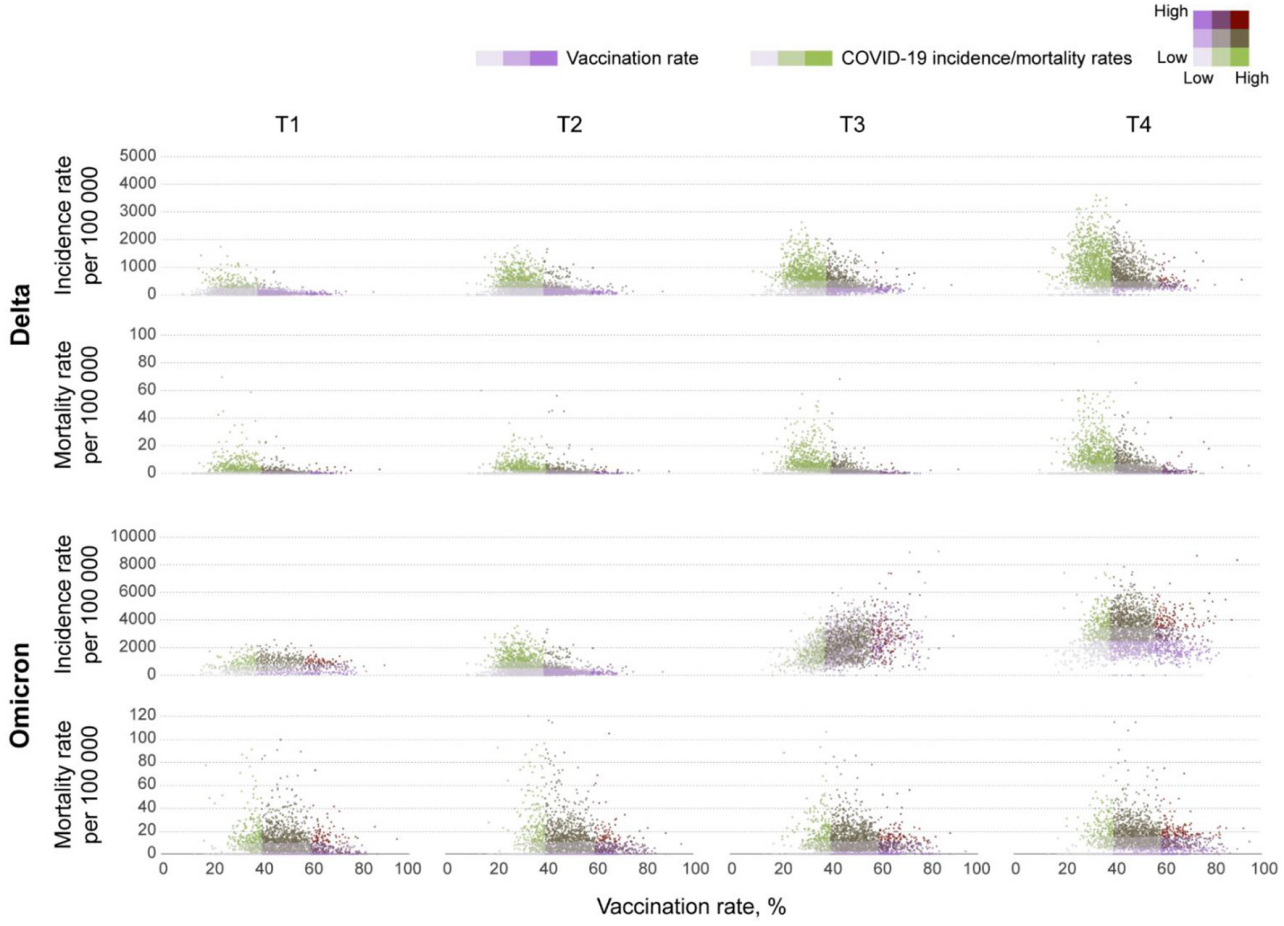
Bivariate scatterplots of the association between vaccination rates and new COVID-19 cases and deaths at the county level in the contiguous U.S. Bivariate scatterplots illustrating the changes of the association between vaccination rates and new COVID-19 cases (incidence rate) and deaths (mortality rate) per 100,000 people at the county level during T1 (July 1–15), T2 (July 16–31), T3 (August 1–15), and T4 (August 16–31) in 2021 for the Delta wave, and T1 (December 1–15), T2 (December 16–31) 2021, T3 (January 1–15), and T4 (January 16–31) in 2022 for the Omicron wave.

**Figure 2:**
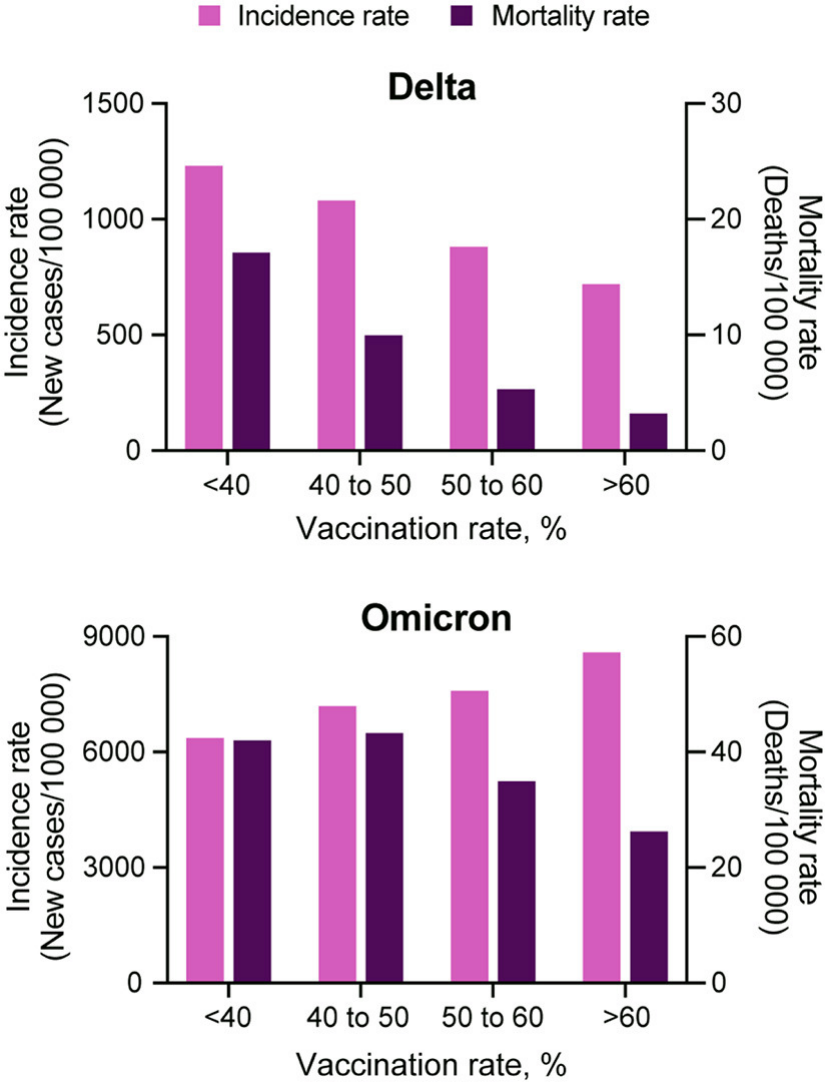
Comparison among COVID-19 incidence and mortality rate and vaccination coverage. COVID-19 incidence (light purple bars) and mortality (dark purple bars) rate estimations for each of the four vaccination rate groups, <40, 40 to 50, 50 to 60% and more than 60%, during the Delta (July 1, 2021 to August 31, 2021) and Omicron (December 1, 2021 to January 31, 2022) waves in the US.

The incidence rate (new COVID-19 cases per 100,000 people) during the Omicron wave increased from 538.9 (95% CI 538.0–539.8) on T1, to 2,827.9 (95% CI 2,825.9–2,829.9) on T4, whereas the mortality rate (COVID-19 related deaths per 100,000 people) increased from 6.1 (95% CI 6.0–6.2) to 11.2 (95% CI 11.1–11.4) during the same period. Scatterplots in Figure 1 illustrate the changes in incidence and mortality rates during the Delta and Omicron waves during the four-time periods of the study (T1–T4). Contrary to the pattern observed during the Delta wave (first row in Figure 1), areas with higher vaccination rates (>60%) experienced the higher and more intense spread of the disease during the Omicron wave (third row in Figure 1). During this wave, the incidence rate was similar in all vaccination rate areas during T1 (December 1 to 15). The infection started spreading faster and more intensively in high vaccination areas during T2 (December 16 to 31) and T3 (January 1 to 15). The incidence rate increased in all vaccination rate areas by T4 (January 16 to 31). It was 1.4 (95% CI 1.3–1.5) times higher in high vaccination rate areas than the lower vaccination areas during the entire period of the study. Conversely, the mortality rate had similar patterns during both the Delta (second row in Figure 1) and the Omicron wave (fourth row in Figure 1), with areas with lower vaccination rate (<40%) having 1.6 (95% CI 1.5–1.7) higher mortality rate compared to areas with higher vaccination rate (>60%), 42.0 (95% CI 40.1–43.2) per 100,000 people in lower vaccination areas, compared to 26.2 (95% CI 25.9–26.5) in higher vaccination areas during the entire period of the study of the Omicron wave. The compiled data for both waves show a clear uncoupling of the vaccination rate and the incidence rate for Omicron while preserving an association with the mortality rate (Figure 2) bottom bar chart. This contrasts with the association between vaccination rate and the number of cases and deaths observed during the Delta wave (Figure 2) top bar chart.

**Figure 3:**
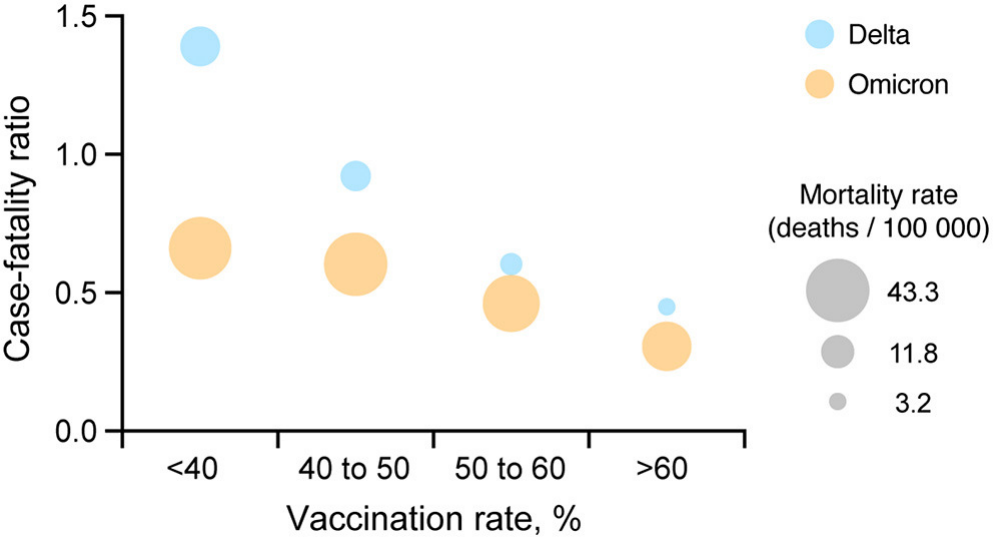
Comparison between COVID-19 case-fatality ratio and vaccination coverage. A multiple variable plot illustrating the COVID-19 case-fatality ratio during the Delta (July 1, 2021 to August 31, 2021; blue circles) and Omicron (December 1, 2021 to January 31, 2022; orange circles) waves for each of the four vaccination rate groups <40, 40 to 50, 50 to 60% and more than 60% in the US.

Comparing incidence and mortality rates values between the two waves needs to take into account that the large number of cases observed during the Omicron wave relative to Delta. For this, we calculated and compared the case-fatality ratio for each vaccination group. Case-fatality ratio estimations illustrated similar patterns during the Delta and Omicron waves (Figure 3), with a consistent reduction of the case-fatality ratio with an increasing vaccination rate. Areas with low vaccination rate had a 2.2 (95% CI 2.1–2.2) higher case-fatality ratio compared to areas with higher vaccination rate during the Omicron wave (0.7% case fatality rate in areas with vaccination rate <40%, and 0.3% in areas with vaccination rate > 60%).

**Figure 4:**
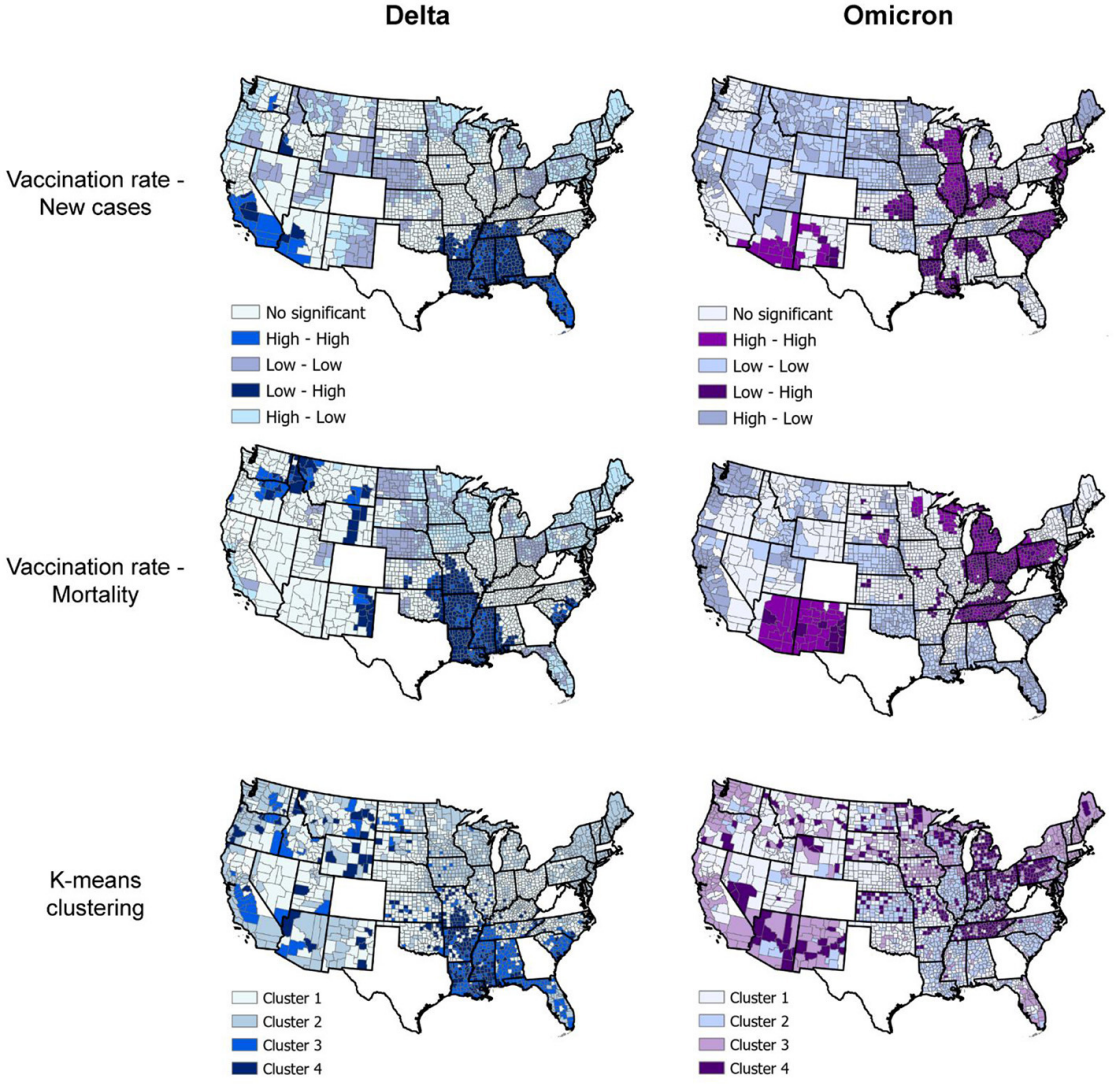
Geospatial clustering of COVID-19 incidence and mortality rates. Bivariate local indicators of spatial autocorrelation and K-means clustering analysis maps for the association between vaccination rate and new COVID-19 cases and deaths per 100,000 people during the Delta (July 1, 2021, to August 31, 2021; blue maps) and Omicron (December 1, 2021 to January 31, 2022; purple maps) in the US. K-means cluster information is summarized in Table 1.

Spatial analyses identified a distinct spatial structure of the pandemic during each wave. The southern part of the country was the epicenter of the Delta wave, whereas the Northeast and Midwest regions experiencing the highest burden of the Omicron wave. Consistent with the results of the incidence rate by vaccination rate group, bivariate LISA analysis identified clusters of low vaccination/high infection rate during the Delta wave in Southern states like Alabama, Arkansas, Louisiana, Mississippi, and Missouri, areas that also suffered high mortality rate during this wave (dark blue areas in the first two maps on top, Figure 4). During the Omicron wave, clusters of high infection rates were concentrated in areas with high vaccination rates in Northeast and Midwest states like Connecticut, New Jersey, Rhode Island, parts of Ohio and Indiana, Illinois, and Wisconsin. Clusters of mortality were not identified in Connecticut, Rhode Island, and New Jersey (areas with high vaccination rates). These clusters moved more to the Midwest and mostly affected states like Pennsylvania, Ohio, Kentucky, Tennessee, and Indiana (dark purple areas in the first two maps on the bottom, Figure 4). Another cluster of high mortality rates was identified in the Western part of the country, including Arizona and New Mexico.

Multivariable K-means clustering analysis illustrated the disease's more defined spatial structure during the Delta compared to the Omicron wave. Clusters 3 and 4 (light and dark blue in the upper right map, Figure 4), both with the lowest vaccination rate (<36%), had the highest incidence and mortality rate (Table 1) and were located in the southern states previously identified by the LISA analysis. Conversely, multivariable K-means clustering analysis showed a more dispersed and less structured distribution of the clusters of these three variables, with Cluster 4 (Dark purple areas in lower right map, Figure 4) primarily located in the states of Tennessee, Pennsylvania, Ohio, Indiana, and Michigan having the highest mortality rates (91.5 deaths per 100,000 people) and the lowest vaccination rates (44.9%). Most of the areas within these clusters with low vaccination and high mortality rates for Delta and Omicron waves were rural. Rural areas corresponded to 73.4% (186 of 252 counties) in Cluster 4 during the Delta wave and 75.4% (355 of 471 counties) in Cluster 4 during the Omicron wave. The percentage of areas with vaccination rate lower than 40% that were rural increased from 75.2% (956 of 1,271 counties) during the Delta wave to 81.6% (421 of 516 counties) during the Omicron wave, and the percentage of areas with vaccination rate lower than 50% that were rural increased from 69.9% (1,377 of 1,970 counties) during the Delta wave to 75.8% (1,029 of 1,358 counties) during the Omicron wave.

**Table 1:** K-means clustering estimations for the Delta (July 1, 2021 to August 31, 2021) and Omicron (December 1, 2021 to January 31, 2022) waves in the US.

	**Cluster**	**Vaccination rate, %**	**Incidence** **rate**	**Mortality** **rate**
Delta	1	34.6	623.5	8.7
	2	52.1	614.1	5.7
	3	35.4	2,404.6	16.9
	4	34.2	1,038.6	51.8
Omicron	1	41.0	4,760.3	19.8
	2	47.6	8,932.0	35.8
	3	62.8	6,883.4	29.8
	4	44.9	6,188.4	91.5

*Cluster locations are illustrated in maps in Figure 4*.

## Discussion

The dynamics of the COVID-19 pandemic have been modulated by control interventions (i.e., non-pharmaceutical interventions and vaccine rollout) and new variants with different transmission dynamics. This has defined the trajectory of the disease, which has been characterized by waves of outbreak peaks, followed by valleys after infection declines. The COVID-19 vaccines developed so far have proven to be the most effective intervention to reduce disease outcomes (hospitalizations and deaths). In an earlier study, we showed that vaccination rollout in the US could influence the epidemiological landscape of the third wave driven by the Delta variant. We also showed that areas with lower vaccination coverage experienced the highest burden of the disease and had 2.4 times higher cases than those with higher vaccination coverage (9). The emergence of the new Omicron variant identified in late 2021 posed a new public health challenge. Omicron is characterized by higher transmissibility conferred by its ability to evade the immune response generated by vaccination or by the previous infection compared to the Delta variant (19, 20), and its ability to evade immune response made this variant responsible for the highest number of breakthrough cases ever reported and changed the virus' scenario compared to the Delta variant.

This study found that contrary to the patterns observed during the Delta wave, highly vaccinated areas in the US experienced the highest burden of new infections during the Omicron wave. Areas with a higher than 60% vaccination rate had 1.4 times more new infections than areas with lower vaccination rates (<40%). This could be explained by the combination of high transmissibility and high population density since most of the areas with high vaccination coverage are urban areas (65%), facilitating transmission (21, 22). However, despite incidence being more prominent in highly vaccinated areas, a completely different result was observed for Omicron related mortality. COVID-19 related mortality rate was 60% higher in areas with low vaccination rate (<40%) compared to areas with high vaccination coverage (>60%). Hence, the case-fatality ratio was more than 2 times higher in low vaccination coverage areas compared with areas with more than 60% vaccination rate, following a similar pattern to that observed for Delta. These results are aligned with other studies conducted in several countries in Europe and the rest of the world, in which the rapid growth of the number of COVID-19 cases has occurred even in countries with high levels of vaccinations. A lower case-fatality ratio in these highly vaccinated countries highlights the fact that although available vaccines cannot prevent new infections or virus spread, they have a high impact on the number of COVID-19 related deaths per capita (23–25).

By comparing the dynamics of the Delta and Omicron's waves, it becomes evident how the different strategies to escape immune response evolved by these two variants have an apparent effect on the epidemiological landscape (26). This new feature in the fusion machinery of Omicron might result in a more efficient infection which can account for the high transmissibility observed in highly populated urban areas despite the high vaccination rate in these areas. The epicenter of the Omicron wave might have started in densely populated and highly connected regions of the Northeast and Midwest in Connecticut, New Jersey, Rhode Island, and Illinois, with large international airports that facilitated the entrance and propagation of this highly infectious variant, a similar pattern observed during the early stages of the pandemic, when vaccines were not available, and large metropolitan areas of the country suffered the highest burden of both COVID-19 infections and deaths (27). However, the introduction of vaccines changed this scenario during the Omicron wave, and although areas with a high percentage of the population fully vaccinated experienced the highest burden of infections product of the high transmissibility and Omicron's ability to evade the immune response, the high vaccination rate prevented a high COVID-19 related mortality in these areas. Conversely, while low vaccination areas had lower transmission intensity, these areas suffered the most increased mortality and case-fatality rate during the Omicron wave in the US, primarily in rural areas (81%).

Despite the lower transmission intensity experienced in lower vaccinated and rural areas, these areas suffered the highest COVID-19 related mortality during the Omicron wave. Low vaccinated areas also experienced the highest burden of cases and deaths during the Delta wave and comparing the percentage change of rural areas with low vaccination rate (<40%) during both waves, vaccination rollout appears to be slower in these rural areas, with the percentage of areas with vaccination coverage lower than 50% that are rural increasing from about 70% during the Delta wave to more than 75% during the Omicron wave. Collectively, these results underscore the persistent vulnerability of the communities residing in these rural areas and highlight the importance of the intensification of vaccination campaigns in these low vaccinated areas. Rural communities often face many challenges that exacerbate health disparities in the country. These areas usually run short on resources, including limited cold chain vaccine storage facilities and healthcare workers to administer vaccines. The geography can also compound disparities in access that affect rural clinics, which face unique challenges to provide vaccinations to residents who live many miles away (28). Moreover, it has been shown that rural residents are less likely to receive flu shots than residents of urban areas (29). This tendency, combined with the hesitancy in rural areas to adopt other COVID-19 mitigation measures, can hamper vaccination efforts in these communities. Furthermore, many of the risk factors for COVID-19 infection complications and deaths are exacerbated in rural areas, particularly in older adults (30, 31). Rural populations are older and have lower general health conditions than urban populations. Therefore, they are vulnerable populations at higher risk of COVID-19 related hospitalization and deaths, with an estimated 10% higher hospitalization rate for COVID-19 per capita than urban residents given equal infection rates (32). Additionally, significant vulnerabilities in rural areas include fewer physicians and lack of access to intensive care and ventilators, which are critical aspects of care needed for at least 5% of critical COVID-19 infection-related complications (33). These combined factors can worsen disease outcomes and influence the higher mortality observed in rural communities during the past two pandemic waves.

Our study had limitations worth noting. An ecological study like the one presented here is an approach for examining the association between factors and diseases by analyzing at the population level in specific areas. Therefore, due to the lack of individual data in ecological studies, it is difficult to adjust for all potential confounding factors. Moreover, there are other factors that might impact the regional incidence and mortality rates including local disparities in healthcare capacity, effectiveness of non-pharmaceutical interventions, COVID-19 testing coverage, and even in data collection and reporting. Lastly, vaccination coverage was estimated using the definition of fully vaccinated individuals, and we did not include data for boosted vaccination. However, by February 2022, boosted vaccination coverage is still lower in the country (~25%) and follows the same spatial pattern of full vaccination coverage.

In conclusion, in this study we found that communities residing in low vaccinated areas are suffering the highest burden of the COVID-19 pandemic during the vaccination era. Despite the emergence of new virus variants with differential transmission potential, the protective effect of vaccines has generated marked differences in the distribution of critical health outcomes, with low vaccinated areas having the largest COVID-19 related mortality during the Delta and Omicron waves. These areas are the most vulnerable even when communities residing in these low vaccinated areas experience lower infection intensity than other areas, which was the epidemiological scenario observed during the Omicron wave. Healthcare disparities and differential vaccination coverage might continue influencing the pandemic trajectory and holding back efforts for epidemic control. Therefore, public information campaigns and vaccine promotions along with the setup of new sites for vaccinations in rural underserved areas should be intensified to target vulnerable populations in rural communities. A successful containment of the epidemic can only be achieved if vaccination intensity is substantially increased to protect the vulnerable population in these underserved areas to diminish the spatial heterogeneity of vaccination in the country. Pandemic control might become a reality only if no one is left behind, not only at local but also at global scale.

## Data Availability Statement

Publicly available datasets were analyzed in this study. This data can be found here: Johns Hopkins Coronavirus Resource Center repository (https://coronavirus.jhu.edu/map.html), and the Centers for Disease Control and Prevention COVID data tracker repository (https://data.cdc.gov/Vaccinations/COVID-19-Vaccinations-in-the-United-States-County/8xkx-amqh).

## Author Contributions

DC and CM: drafting of the manuscript and statistical analysis. All authors: concept and design, acquisition, analysis, interpretation of data, and critical revision of the manuscript for important intellectual content.

## Conflict of Interest

The authors declare that the research was conducted in the absence of any commercial or financial relationships that could be construed as a potential conflict of interest.

## Publisher's Note

All claims expressed in this article are solely those of the authors and do not necessarily represent those of their affiliated organizations, or those of the publisher, the editors and the reviewers. Any product that may be evaluated in this article, or claim that may be made by its manufacturer, is not guaranteed or endorsed by the publisher.
